# *N*,*N*′-(4,5-Dimethyl-1,2-phenyl­ene)bis­(pyridine-2-carboxamide)

**DOI:** 10.1107/S1600536812035064

**Published:** 2012-08-23

**Authors:** Phillipus C. W. Van der Berg, Hendrik G. Visser, Andreas Roodt, Theunis J. Muller

**Affiliations:** a Department of Chemistry, University of the Free State, PO Box 339, Bloemfontein 9300, South Africa

## Abstract

In the title compound, C_20_H_18_N_4_O_2_, the dihedral angles between the central benzene ring and the pyridine rings are 57.55 (6) and 22.05 (8)°. The mol­ecular conformation is stabilized by intra­molecular N—H⋯N inter­actions and in the crystal structure an inter­molecular asymmetric cyclic hydrogen-bonding association involving both amide N—H donors and a common amide O-atom acceptor gives a chain extending along the *c* axis.

## Related literature

For related structures, see: Jain *et al.* (2004 ▶); Lin *et al.* (2001 ▶); Roodt *et al.* (2011 ▶); Schutte *et al.* (2011 ▶); Van der Berg *et al.* (2011 ▶).
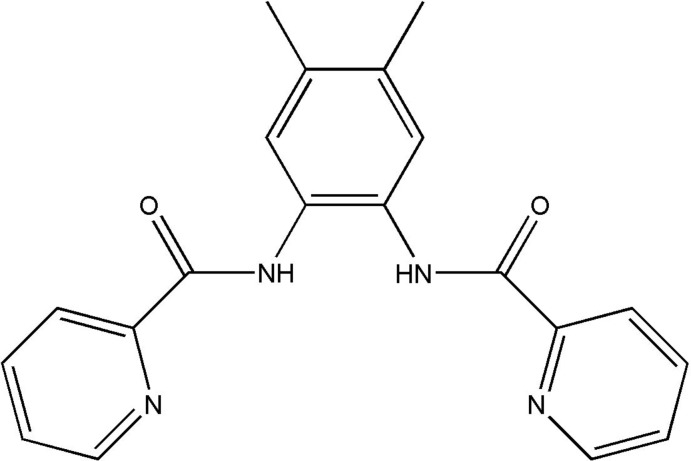


## Experimental

### 

#### Crystal data

C_20_H_18_N_4_O_2_
*M* *_r_* = 346.38
Monoclinic, 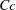

*a* = 12.1299 (8) Å
*b* = 18.9418 (8) Å
*c* = 7.7549 (4) Å
β = 100.375 (4)°
*V* = 1752.65 (17) Å^3^
*Z* = 4
Mo *K*α radiation
μ = 0.09 mm^−1^
*T* = 100 K
0.78 × 0.08 × 0.07 mm

#### Data collection

Bruker X8 APEXII KappaCCD diffractometer
Absorption correction: multi-scan (*SADABS*; Bruker, 2004 ▶) *T*_min_ = 0.990, *T*_max_ = 0.994
15674 measured reflections
3860 independent reflections
3529 reflections with *I* > 2σ(*I*)
*R*_int_ = 0.031

#### Refinement

*R*[*F* ^2^ > 2σ(*F* ^2^)] = 0.036
*wR*(*F* ^2^) = 0.090
*S* = 1.04
3860 reflections
237 parameters
2 restraints
H atoms treated by a mixture of independent and constrained refinement
Δρ_max_ = 0.26 e Å^−3^
Δρ_min_ = −0.20 e Å^−3^

### 

Data collection: *APEX2* (Bruker, 2010 ▶); cell refinement: *SAINT-Plus* (Bruker, 2004 ▶); data reduction: *SAINT-Plus*; program(s) used to solve structure: *SHELXS97* (Sheldrick, 2008 ▶); program(s) used to refine structure: *SHELXL97* (Sheldrick, 2008 ▶); molecular graphics: *DIAMOND* (Brandenburg & Putz, 2005 ▶); software used to prepare material for publication: *WinGX* (Farrugia, 1999 ▶).

## Supplementary Material

Crystal structure: contains datablock(s) global, I. DOI: 10.1107/S1600536812035064/zs2225sup1.cif

Structure factors: contains datablock(s) I. DOI: 10.1107/S1600536812035064/zs2225Isup2.hkl

Additional supplementary materials:  crystallographic information; 3D view; checkCIF report

## Figures and Tables

**Table 1:** Hydrogen-bond geometry (Å, °)

*D*—H⋯*A*	*D*—H	H⋯*A*	*D*⋯*A*	*D*—H⋯*A*
N2—H2′⋯N1	0.86 (2)	2.20 (2)	2.6698 (19)	114.2 (16)
N2—H2′⋯N3	0.86 (2)	2.48 (2)	2.777 (2)	101.2 (15)
N2—H2′⋯O2^i^	0.86 (2)	2.60 (2)	3.2112 (19)	129.0 (17)
N3—H3′⋯O2^i^	0.86 (2)	2.05 (2)	2.8508 (19)	155.5 (18)
N3—H3′⋯N4	0.86 (2)	2.28 (2)	2.6670 (19)	107.8 (15)

Symmetry code: (i) 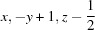.

**Fig. 1.**
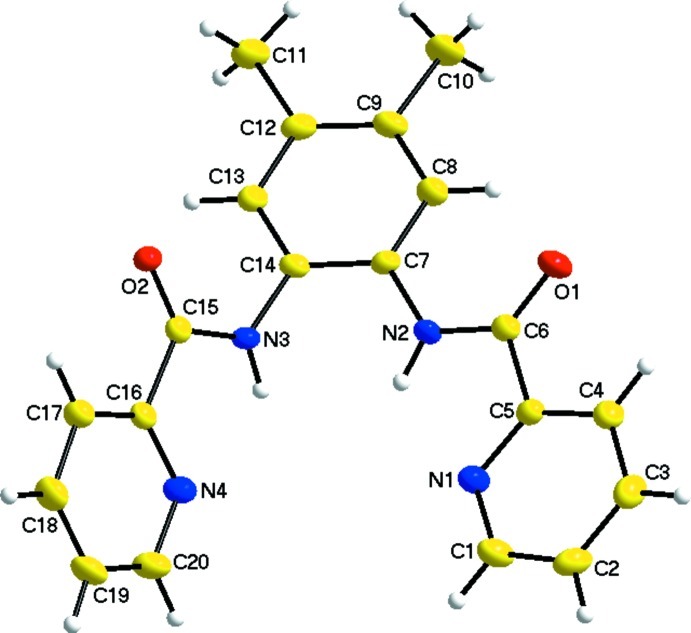
Molecular structure of the title compound, showing the atom numbering scheme, with displacement ellipsoids drawn at the 50% probability level.

## supplementary crystallographic information

### Comment

The title compound C_20_H_18_N_4_O_2_ was synthesized as a ligand for potential use in medical and radiopharmaceutical applications. In this compound, which has one molecule in the asymmetric unit (Fig. 1), the dihedral angles between the central benzene ring and the pyridine rings are 57.55 (6) and 22.05 (8)°. The molecular conformation is stabilized by intramolecular N—H···N interactions and in the crystal structure an intermolecular asymmetric cyclic hydrogen-bonding association involving both amide N—H donors and a common amide O-atom acceptor (O2^i^) (Table 1), give a one-dimensional chain extending along *c*. The related structures from Roodt *et al.* (2011) and Schutte *et al.* (2011) also contribute to our studies in radiopharmaceutical design and reactivity.

### Experimental

Under oxygen atmosphere, picolinic acid (5.73 g, 0.0465 mol) was added as a solid in one portion to a suspension of 4,5-dimethyl-1,2-phenylenediamine (3.00 g, 0.0220 mol) in pyridine (20 ml) and the mixture was stirred at 40 °C for 40 min. Triphenylphosphite (30 ml) was added dropwise over 10 minutes after which the temperature was increased to 90–100 °C and stirred for a further 24 h. On cooling the precipitate was filtered, washed with H_2_O (50 ml) and then MeOH (50 ml). The precipitate was recrystallized in chloroform to giving colourless crystals after five days

### Refinement

The amides, aromatic and methyl hydrogen atoms were positioned geometrically and allowed to ride on their parent atoms, N—H = 0.86 Å and *U*_iso_(H) = 1.2*U*_eq_, C—H (aromatic C) = 0.95 Å and *U*_iso_(H) = 1.2*U*_eq_ and C—H (methyl C) = 0.98 Å and *U*_iso_(H) = 1.5*U*_eq_ respectively. The methyl groups were allowed to rotate, giving six half-H sites.

### Crystal data

**Table:**

C_20_H_18_N_4_O_2_	*F*(000) = 728
*M**_r_* = 346.38	*D*_x_ = 1.313 Mg m^−^^3^
Monoclinic, *C**c*	Mo *K*α radiation, λ = 0.71073 Å
Hall symbol: C -2yc	Cell parameters from 5487 reflections
*a* = 12.1299 (8) Å	θ = 3.1–28.3°
*b* = 18.9418 (8) Å	µ = 0.09 mm^−^^1^
*c* = 7.7549 (4) Å	*T* = 100 K
β = 100.375 (4)°	Needle, colourless
*V* = 1752.65 (17) Å^3^	0.78 × 0.08 × 0.07 mm
*Z* = 4	

### Data collection

**Table:**

Bruker X8 APEXII KappaCCD diffractometer	3860 independent reflections
Radiation source: sealed tube	3529 reflections with *I* > 2σ(*I*)
Graphite monochromator	*R*_int_ = 0.031
φ and ω scans	θ_max_ = 28°, θ_min_ = 3.1°
Absorption correction: multi-scan (*SADABS*; Bruker, 2004)	*h* = −16→16
*T*_min_ = 0.990, *T*_max_ = 0.994	*k* = −24→24
15674 measured reflections	*l* = −10→10

### Refinement

**Table:**

Refinement on *F*^2^	Primary atom site location: structure-invariant direct methods
Least-squares matrix: full	Secondary atom site location: difference Fourier map
*R*[*F*^2^ > 2σ(*F*^2^)] = 0.036	Hydrogen site location: inferred from neighbouring sites
*wR*(*F*^2^) = 0.090	H atoms treated by a mixture of independent and constrained refinement
*S* = 1.04	*w* = 1/[σ^2^(*F*_o_^2^) + (0.0456*P*)^2^ + 0.7043*P*] where *P* = (*F*_o_^2^ + 2*F*_c_^2^)/3
3860 reflections	(Δ/σ)_max_ = 0.001
237 parameters	Δρ_max_ = 0.26 e Å^−^^3^
2 restraints	Δρ_min_ = −0.20 e Å^−^^3^

### Special details

**Table:**

Experimental. The intensity data was collected on a Bruker X8 ApexII 4 K Kappa CCD diffractometer using an exposure time of 30 s/frame. A total of 1895 frames was collected with a frame width of 0.5° covering up to θ = 28.29° with 99.9% completeness accomplished.
Geometry. All s.u.'s (except the s.u. in the dihedral angle between two l.s. planes) are estimated using the full covariance matrix. The cell s.u.'s are taken into account individually in the estimation of s.u.'s in distances, angles and torsion angles; correlations between s.u.'s in cell parameters are only used when they are defined by crystal symmetry. An approximate (isotropic) treatment of cell s.u.'s is used for estimating s.u.'s involving l.s. planes.
Refinement. Refinement of *F*^2^ against ALL reflections. The weighted *R*-factor *wR* and goodness of fit *S* are based on *F*^2^, conventional *R*-factors *R* are based on *F*, with *F* set to zero for negative *F*^2^. The threshold expression of *F*^2^ > 2σ(*F*^2^) is used only for calculating *R*-factors(gt) *etc*. and is not relevant to the choice of reflections for refinement. *R*-factors based on *F*^2^ are statistically about twice as large as those based on *F*, and *R*- factors based on ALL data will be even larger.

### Fractional atomic coordinates and isotropic or equivalent isotropic displacement parameters (Å^2^)

**Table:**

	*x*	*y*	*z*	*U*_iso_*/*U*_eq_	Occ. (<1)
C6	0.77131 (13)	0.70520 (9)	0.3849 (2)	0.0204 (3)	
C10	0.34827 (15)	0.64314 (11)	0.4195 (3)	0.0336 (4)	
H10A	0.2857	0.6104	0.4193	0.050*	0.5
H10B	0.3246	0.6815	0.3361	0.050*	0.5
H10C	0.3713	0.6629	0.5373	0.050*	0.5
H10D	0.3688	0.6927	0.4424	0.050*	0.5
H10E	0.3298	0.6217	0.5257	0.050*	0.5
H10F	0.2831	0.6403	0.3245	0.050*	0.5
C11	0.33503 (15)	0.48994 (11)	0.3373 (3)	0.0313 (4)	
H11A	0.3459	0.4411	0.3025	0.047*	0.5
H11B	0.2703	0.5102	0.2592	0.047*	0.5
H11C	0.3218	0.4908	0.4583	0.047*	0.5
H11D	0.2794	0.5204	0.3775	0.047*	0.5
H11E	0.355	0.4512	0.4208	0.047*	0.5
H11F	0.3035	0.4706	0.2217	0.047*	0.5
C15	0.77150 (13)	0.44582 (8)	0.2917 (2)	0.0202 (2)	
N2	0.73461 (11)	0.64533 (7)	0.2985 (2)	0.0221 (3)	
N3	0.72056 (11)	0.50299 (7)	0.21172 (19)	0.0190 (3)	
O1	0.72183 (10)	0.73844 (7)	0.48200 (17)	0.0277 (3)	
O2	0.74071 (9)	0.41395 (6)	0.41242 (15)	0.0202 (2)	
H2'	0.7803 (17)	0.6283 (10)	0.237 (3)	0.027 (5)*	
H3'	0.7438 (16)	0.5199 (10)	0.122 (3)	0.024 (5)*	
C1	1.03548 (14)	0.71236 (10)	0.2222 (2)	0.0283 (4)	
H1	1.0731	0.6853	0.1474	0.034*	
C2	1.08852 (15)	0.77174 (10)	0.3009 (2)	0.0293 (4)	
H2	1.1603	0.7852	0.2795	0.035*	
C3	1.03552 (16)	0.81103 (10)	0.4107 (2)	0.0289 (4)	
H3	1.07	0.8521	0.4668	0.035*	
C4	0.93064 (15)	0.78956 (9)	0.4380 (2)	0.0249 (4)	
H4	0.8918	0.8154	0.5134	0.03*	
C5	0.88403 (13)	0.72965 (9)	0.3529 (2)	0.0197 (3)	
C7	0.63512 (12)	0.60847 (8)	0.3093 (2)	0.0192 (3)	
C8	0.54288 (15)	0.64111 (9)	0.3586 (2)	0.0241 (3)	
H8	0.5468	0.6899	0.3874	0.029*	
C9	0.44549 (13)	0.60415 (9)	0.3667 (2)	0.0243 (4)	
C12	0.43827 (14)	0.53253 (9)	0.3249 (2)	0.0235 (3)	
C13	0.53017 (14)	0.50011 (9)	0.2728 (2)	0.0215 (3)	
H13	0.5258	0.4515	0.2421	0.026*	
C14	0.62761 (12)	0.53699 (8)	0.2646 (2)	0.0189 (3)	
C16	0.87530 (13)	0.42368 (9)	0.2250 (2)	0.0189 (3)	
C17	0.93911 (15)	0.36937 (9)	0.3085 (2)	0.0255 (4)	
H17	0.9157	0.344	0.4012	0.031*	
C18	1.03894 (15)	0.35268 (10)	0.2530 (3)	0.0295 (4)	
H18	1.0859	0.316	0.3079	0.035*	
C19	1.06792 (15)	0.39063 (10)	0.1168 (2)	0.0283 (4)	
H19	1.1357	0.3806	0.0764	0.034*	
C20	0.99767 (15)	0.44332 (10)	0.0395 (3)	0.0301 (4)	
H20	1.0181	0.4685	−0.056	0.036*	
N1	0.93415 (12)	0.69083 (8)	0.2456 (2)	0.0248 (3)	
N4	0.90251 (12)	0.46069 (8)	0.09200 (19)	0.0245 (3)	

### Atomic displacement parameters (Å^2^)

**Table:**

	*U*^11^	*U*^22^	*U*^33^	*U*^12^	*U*^13^	*U*^23^
C6	0.0205 (7)	0.0210 (8)	0.0200 (8)	0.0032 (6)	0.0050 (6)	0.0032 (6)
C10	0.0211 (8)	0.0387 (10)	0.0437 (12)	0.0041 (7)	0.0132 (8)	−0.0001 (9)
C11	0.0227 (8)	0.0378 (10)	0.0351 (10)	−0.0060 (7)	0.0102 (8)	−0.0020 (8)
C15	0.0209 (4)	0.0201 (5)	0.0205 (4)	−0.0022 (3)	0.0064 (4)	−0.0014 (4)
N2	0.0184 (7)	0.0206 (7)	0.0304 (8)	0.0008 (5)	0.0124 (6)	−0.0027 (6)
N3	0.0181 (6)	0.0214 (7)	0.0192 (7)	−0.0007 (5)	0.0081 (5)	0.0004 (6)
O1	0.0252 (6)	0.0322 (7)	0.0283 (7)	0.0007 (5)	0.0117 (5)	−0.0061 (6)
O2	0.0209 (4)	0.0201 (5)	0.0205 (4)	−0.0022 (3)	0.0064 (4)	−0.0014 (4)
C1	0.0238 (8)	0.0362 (10)	0.0265 (9)	−0.0018 (7)	0.0088 (7)	−0.0063 (8)
C2	0.0253 (9)	0.0384 (11)	0.0253 (9)	−0.0092 (7)	0.0078 (7)	0.0008 (8)
C3	0.0343 (10)	0.0279 (9)	0.0243 (9)	−0.0102 (7)	0.0043 (8)	−0.0031 (8)
C4	0.0291 (9)	0.0253 (8)	0.0215 (8)	−0.0022 (7)	0.0079 (7)	−0.0008 (7)
C5	0.0196 (7)	0.0191 (8)	0.0213 (8)	0.0005 (6)	0.0061 (6)	0.0034 (6)
C7	0.0168 (7)	0.0215 (8)	0.0202 (8)	−0.0008 (6)	0.0060 (6)	0.0027 (6)
C8	0.0212 (7)	0.0253 (8)	0.0268 (9)	0.0037 (7)	0.0072 (7)	0.0020 (7)
C9	0.0196 (8)	0.0314 (9)	0.0232 (8)	0.0047 (7)	0.0070 (7)	0.0027 (7)
C12	0.0174 (7)	0.0329 (9)	0.0205 (8)	−0.0024 (7)	0.0041 (6)	0.0035 (7)
C13	0.0217 (7)	0.0238 (8)	0.0196 (8)	−0.0022 (6)	0.0055 (6)	−0.0005 (6)
C14	0.0173 (7)	0.0240 (8)	0.0158 (8)	0.0020 (6)	0.0044 (6)	0.0014 (6)
C16	0.0188 (7)	0.0188 (7)	0.0190 (7)	−0.0015 (6)	0.0035 (6)	−0.0040 (6)
C17	0.0262 (8)	0.0248 (8)	0.0274 (9)	0.0022 (7)	0.0097 (7)	0.0020 (7)
C18	0.0271 (9)	0.0278 (9)	0.0346 (10)	0.0079 (7)	0.0079 (8)	0.0012 (8)
C19	0.0210 (8)	0.0356 (10)	0.0302 (9)	0.0049 (7)	0.0096 (7)	−0.0046 (8)
C20	0.0274 (9)	0.0375 (10)	0.0282 (9)	0.0030 (7)	0.0128 (8)	0.0046 (8)
N1	0.0218 (7)	0.0269 (7)	0.0275 (8)	−0.0029 (6)	0.0089 (6)	−0.0051 (6)
N4	0.0231 (7)	0.0290 (8)	0.0227 (7)	0.0031 (6)	0.0080 (6)	0.0027 (6)

### Geometric parameters (Å, º)

**Table:**

C6—O1	1.219 (2)	C1—H1	0.95
C6—N2	1.351 (2)	C2—C3	1.375 (3)
C6—C5	1.506 (2)	C2—H2	0.95
C10—C9	1.509 (2)	C3—C4	1.388 (2)
C10—H10A	0.98	C3—H3	0.95
C10—H10B	0.98	C4—C5	1.381 (2)
C10—H10C	0.98	C4—H4	0.95
C10—H10D	0.98	C5—N1	1.336 (2)
C10—H10E	0.98	C7—C8	1.391 (2)
C10—H10F	0.98	C7—C14	1.397 (2)
C11—C12	1.507 (2)	C8—C9	1.384 (2)
C11—H11A	0.98	C8—H8	0.95
C11—H11B	0.98	C9—C12	1.394 (2)
C11—H11C	0.98	C12—C13	1.395 (2)
C11—H11D	0.98	C13—C14	1.384 (2)
C11—H11E	0.98	C13—H13	0.95
C11—H11F	0.98	C16—N4	1.336 (2)
C15—O2	1.2274 (19)	C16—C17	1.377 (2)
C15—N3	1.342 (2)	C17—C18	1.392 (2)
C15—C16	1.505 (2)	C17—H17	0.95
N2—C7	1.410 (2)	C18—C19	1.374 (3)
N2—H2'	0.86 (2)	C18—H18	0.95
N3—C14	1.421 (2)	C19—C20	1.378 (3)
N3—H3'	0.86 (2)	C19—H19	0.95
C1—N1	1.338 (2)	C20—N4	1.333 (2)
C1—C2	1.382 (3)	C20—H20	0.95
			
O1—C6—N2	125.78 (16)	C15—N3—H3'	119.0 (13)
O1—C6—C5	120.35 (15)	C14—N3—H3'	117.3 (13)
N2—C6—C5	113.87 (14)	N1—C1—C2	123.65 (17)
C9—C10—H10A	109.5	N1—C1—H1	118.2
C9—C10—H10B	109.5	C2—C1—H1	118.2
H10A—C10—H10B	109.5	C3—C2—C1	118.80 (16)
C9—C10—H10C	109.5	C3—C2—H2	120.6
H10A—C10—H10C	109.5	C1—C2—H2	120.6
H10B—C10—H10C	109.5	C2—C3—C4	118.66 (16)
C9—C10—H10D	109.5	C2—C3—H3	120.7
H10A—C10—H10D	141.1	C4—C3—H3	120.7
H10B—C10—H10D	56.3	C5—C4—C3	118.40 (16)
H10C—C10—H10D	56.3	C5—C4—H4	120.8
C9—C10—H10E	109.5	C3—C4—H4	120.8
H10A—C10—H10E	56.3	N1—C5—C4	123.82 (14)
H10B—C10—H10E	141.1	N1—C5—C6	117.49 (14)
H10C—C10—H10E	56.3	C4—C5—C6	118.67 (15)
H10D—C10—H10E	109.5	C8—C7—C14	118.66 (14)
C9—C10—H10F	109.5	C8—C7—N2	122.39 (15)
H10A—C10—H10F	56.3	C14—C7—N2	118.93 (14)
H10B—C10—H10F	56.3	C9—C8—C7	121.53 (15)
H10C—C10—H10F	141.1	C9—C8—H8	119.2
H10D—C10—H10F	109.5	C7—C8—H8	119.2
H10E—C10—H10F	109.5	C8—C9—C12	120.01 (15)
C12—C11—H11A	109.5	C8—C9—C10	118.66 (16)
C12—C11—H11B	109.5	C12—C9—C10	121.33 (16)
H11A—C11—H11B	109.5	C9—C12—C13	118.41 (15)
C12—C11—H11C	109.5	C9—C12—C11	121.62 (16)
H11A—C11—H11C	109.5	C13—C12—C11	119.96 (16)
H11B—C11—H11C	109.5	C14—C13—C12	121.65 (15)
C12—C11—H11D	109.5	C14—C13—H13	119.2
H11A—C11—H11D	141.1	C12—C13—H13	119.2
H11B—C11—H11D	56.3	C13—C14—C7	119.73 (14)
H11C—C11—H11D	56.3	C13—C14—N3	120.85 (14)
C12—C11—H11E	109.5	C7—C14—N3	119.42 (13)
H11A—C11—H11E	56.3	N4—C16—C17	123.97 (15)
H11B—C11—H11E	141.1	N4—C16—C15	117.22 (14)
H11C—C11—H11E	56.3	C17—C16—C15	118.74 (14)
H11D—C11—H11E	109.5	C16—C17—C18	118.08 (16)
C12—C11—H11F	109.5	C16—C17—H17	121
H11A—C11—H11F	56.3	C18—C17—H17	121
H11B—C11—H11F	56.3	C19—C18—C17	118.40 (17)
H11C—C11—H11F	141.1	C19—C18—H18	120.8
H11D—C11—H11F	109.5	C17—C18—H18	120.8
H11E—C11—H11F	109.5	C18—C19—C20	119.32 (16)
O2—C15—N3	124.85 (15)	C18—C19—H19	120.3
O2—C15—C16	120.91 (14)	C20—C19—H19	120.3
N3—C15—C16	114.22 (14)	N4—C20—C19	123.20 (17)
C6—N2—C7	126.56 (14)	N4—C20—H20	118.4
C6—N2—H2'	113.8 (13)	C19—C20—H20	118.4
C7—N2—H2'	119.5 (13)	C5—N1—C1	116.67 (14)
C15—N3—C14	123.74 (14)	C20—N4—C16	117.01 (15)

### Hydrogen-bond geometry (Å, º)

**Table:**

*D*—H···*A*	*D*—H	H···*A*	*D*···*A*	*D*—H···*A*
N2—H2′···N1	0.86 (2)	2.20 (2)	2.6698 (19)	114.2 (16)
N2—H2′···N3	0.86 (2)	2.48 (2)	2.777 (2)	101.2 (15)
N2—H2′···O2^i^	0.86 (2)	2.60 (2)	3.2112 (19)	129.0 (17)
N3—H3′···O2^i^	0.86 (2)	2.05 (2)	2.8508 (19)	155.5 (18)
N3—H3′···N4	0.86 (2)	2.28 (2)	2.6670 (19)	107.8 (15)
C8—H8···O1	0.95	2.31	2.877 (2)	118
C2—H2···O1^ii^	0.95	2.59	3.195 (2)	122
C3—H3···O2^iii^	0.95	2.48	3.160 (2)	128

Symmetry codes: (i) *x*, −*y*+1, *z*−1/2; (ii) *x*+1/2, −*y*+3/2, *z*−1/2; (iii) *x*+1/2, *y*+1/2, *z*.
